# Duration of Methadone and Buprenorphine-Naloxone Treatment

**DOI:** 10.1001/jamanetworkopen.2025.18389

**Published:** 2025-07-01

**Authors:** Robert A. Kleinman, Paul Kurdyak

**Affiliations:** 1 Institute for Mental Health Policy Research, Centre for Addiction and Mental Health, Toronto, Ontario, Canada; 2 Department of Psychiatry, Faculty of Medicine, University of Toronto, Toronto, Ontario, Canada; 3 Mental Health and Addictions Program, ICES, Toronto, Ontario, Canada

## Abstract

**Importance:**

Fentanyl has spread through the illicit opioid supply in Canada, driving increasing overdose deaths. However, the effectiveness of methadone and buprenorphine-naloxone in treating opioid use disorder during the fentanyl era is unknown.

**Objective:**

To evaluate methadone and buprenorphine-naloxone treatment duration, a core effectiveness outcome in the treatment of opioid use disorder, in Ontario, Canada, between 2014 and 2022.

**Design, Setting, and Participants:**

This population-based, retrospective cohort study included individuals who initiated methadone or buprenorphine-naloxone between January 2014 and December 2022 in Ontario, Canada. Data were analyzed from July 18, 2023, to June 11, 2025.

**Exposure:**

Period of medication initiation (2014-2016, 2017-2019, or 2020-2022).

**Main Outcomes and Measures:**

The main outcome was treatment duration, measured as time to medication discontinuation (5 consecutive days without dispensation of the initial opioid agonist treatment or availability of take-home doses).

**Results:**

The cohort included 72 717 new recipients of opioid agonist treatments (45 256 [62.2%] male; median [IQR] age, 35 [28-46] years), with 34 538 individuals (47.5%) receiving methadone and 38 179 individuals (52.5%) receiving buprenorphine-naloxone. Among individuals starting methadone, median treatment duration decreased from 193 (95% CI, 185-202) days in 2014 to 2016 to 139 (95% CI, 130-149) days in 2017 to 2019 and 86 (95% CI, 78-95) days in 2020 to 2022. Among individuals starting buprenorphine-naloxone, median treatment duration decreased from 51 (95% CI, 49-54) days in 2014 to 2016 and 50 (95% CI, 48-53) days in 2017 to 2019 to 38 (95% CI, 36-40) days in 2020 to 2022. In adjusted Cox regression models including time-varying effects and using 2014 to 2016 as the reference period, hazards of discontinuation measured at treatment initiation were higher during later periods of methadone initiation (2017-2019: adjusted hazard ratio [aHR], 1.18 [95% CI, 1.15-1.22]; *P* < .001; 2020-2022: aHR, 1.45 [95% CI, 1.39-1.51]; *P* < .001) and for buprenorphine-naloxone initiation in 2020 to 2022 (aHR, 1.11 [95% CI, 1.08-1.15]; *P* < .001). Age categories, neighborhood income quintile, rurality, sex, and number of comorbidities were also associated with time to discontinuation in adjusted models.

**Conclusions and Relevance:**

This cohort study found that treatment duration among individuals starting methadone and buprenorphine-naloxone during 2020 to 2022 was lower than during 2014 to 2016. This study highlights the importance of ongoing evaluation of treatment effectiveness, given the dynamic nature of the opioid crisis. Further research is needed to improve treatment retention and improve the effectiveness of opioid use disorder treatment.

## Key Points

**Question:**

Have methadone and buprenorphine-naloxone treatment duration changed between 2014 and 2022 in Ontario, Canada?

**Findings:**

In this cohort study including 72 717 new recipients of opioid agonist treatments, median treatment duration with methadone decreased from 193 days in 2014 to 2016 to 86 days in 2020 to 2022, and median treatment duration with buprenorphine-naloxone decreased from 51 days in 2014 to 2016 to 38 days in 2020 to 2022.

**Meaning:**

Treatment duration decreased during the study period, which coincided with an increase in fentanyl in the illicit opioid supply.

## Teaser

This cohort study evaluates duration of methadone or buprenorphine-naloxone treatment among new recipients of opioid agonist treatments in Ontario, Canada, between 2014 and 2022.

## Introduction

Opioid agonist treatments (OATs), such as methadone and buprenorphine-naloxone, are the main treatments for opioid use disorder (OUD).^1,2^ Among individuals with OUD, the use of methadone and buprenorphine-naloxone has been associated with reductions in opioid overdoses and all-cause mortality.^1,3^ However, most data about the effectiveness of methadone and buprenorphine-naloxone is from studies involving individuals using heroin or prescription opioids.^1,3,4^ Fentanyl, a potent synthetic opioid, has spread throughout the illicit drug supply in Canada, and anecdotal patient and clinician experiences suggest that methadone and buprenorphine-naloxone may be less effective for treating OUD involving fentanyl use.^5,6^

However, beyond anecdotal reports, there are limited data about whether the effectiveness of methadone and buprenorphine-naloxone have changed with the emergence of fentanyl. We conducted a retrospective population-based cohort study evaluating treatment duration among individuals starting methadone and buprenorphine-naloxone as fentanyl entered the illicit opioid supply in Ontario, Canada. Treatment duration is a core outcome for evaluating OUD treatment effectiveness, and studies across jurisdictions have shown increased rates of opioid overdose after methadone and buprenorphine-naloxone discontinuation.^1,3,7,8^

## Methods

This cohort study was approved by the Centre for Addiction and Mental Health Research Ethics Board. The requirement for informed consent was waived under Canada’s Tri-Council Policy Statement 2. Reporting of this study follows the Reporting of Studies Conducted Using Observational Routinely Collected Data for Pharmacoepidemiology Research (RECORD-PE) reporting guidelines.^9^

### Study Design and Data Sources

The study was a population-based, retrospective cohort study. We obtained administrative data for the study from ICES (formerly known as the Institute for Clinical and Evaluative Sciences). ICES is an independent, nonprofit research institute whose legal status under Ontario’s health information privacy law allows it to collect and analyze health care and demographic data, without consent, for health system evaluation and improvement. We obtained information about methadone and buprenorphine-naloxone dispensing, including dates of dispensing and number of take-home doses supplied, through the Narcotic Monitoring System (NMS). Take-home doses are doses of methadone or buprenorphine-naloxone dispensed to an individual for future, unsupervised use. The NMS records information about all dispensing of controlled substances (including methadone and buprenorphine-naloxone) from outpatient pharmacies in Ontario.^10^

Information about baseline characteristics and covariates was obtained from ICES data holdings, including the Discharge Abstract Database, the National Ambulatory Care Reporting System, validated disease-specific cohorts, the Ontario Health Insurance Program Claims Database, and the Same-Day Surgery Database (eTable 1 in Supplement 1). These datasets were linked using unique encoded identifiers and analyzed at ICES.

### Participants

Individuals were included in the cohort if they were an Ontario resident with a new initiation of methadone or buprenorphine-naloxone between January 1, 2014, and December 31, 2022. A new initiation of methadone or buprenorphine-naloxone was defined as the first outpatient dispensing of the medication, with no dispensing of any form of OAT (methadone, buprenorphine-naloxone, buprenorphine–extended release, or slow-release oral morphine) during the prior 365 days. The index date was defined as the day of first dispensing of the medication. Individuals were excluded from the cohort if they had dispensing of more than 1 type of OAT on the index date. Individuals were also excluded from the cohort if they had no Ontario Health Insurance Program coverage (due to an inability to link data across databases), were younger than 15 years, or had a recorded date of death prior to the index date. If individuals had more than 1 treatment episode meeting inclusion and exclusion criteria, only the first treatment episode was included.

### Exposure

The exposure within this study was the time period of medication initiation (2014-2016, 2017-2019, or 2020-2022). These periods correspond to periods of low, increasing, and high fentanyl penetration into the illicit drug supply in Ontario, Canada and increasing involvement of fentanyl in opioid-involved overdose deaths.^5,11^

### Outcome

The outcome of the study was discontinuation of the medication dispensed on the index date. Discontinuation was defined as 5 consecutive nonhospitalized days without a dispensing of the medication and without availability of previously dispensed take-home doses of the medication.

### Baseline Characteristics

Sociodemographic information was obtained about individuals at the time of the index date. We obtained information about medical comorbidities based on health care diagnoses recorded over a 1-year lookback period or inclusion within validated disease-specific cohorts (eTable 1 in Supplement 1). We used the number of Aggregated Diagnosis Groups (ADGs) from the Johns Hopkins’ Adjusted Clinical Groups (version 10) to summarize overall comorbidity burden.

### Follow-Up and Censoring

Individuals were followed until the date of medication discontinuation or censoring. Individuals were censored if they left the province of Ontario, died during study follow-up, switched or were dispensed an additional form of OAT (including slow-release oral morphine and buprenorphine–extended release), or reached the maximum follow-up date of December 31, 2023.

### Statistical Analysis

Descriptive statistics were calculated for individuals starting methadone and buprenorphine-naloxone. We conducted time-to-event analyses, comparing time to discontinuation between individuals in the 3 time periods, stratified by medication type. Median treatment duration was calculated as the time at which 50% of individuals had discontinued treatment, accounting for censoring. We used cause-specific, multivariable Cox proportional hazards models to compare treatment duration across the time periods, adjusting for age at index (as a categorical variable), sex, rurality, neighborhood income quintile, and comorbidities (measured by number of Johns Hopkins ADGs). These covariates were selected given their associations with methadone or buprenorphine-naloxone treatment duration in previous studies.^12,13,14^ We included a covariate for a time × index period interaction, given nonproportionality identified on visual inspection of model log-log plots. Time-to-event analyses were stratified by OAT type. *P* values were 2-sided, and statistical significance was set at *P* ≤ .05. All analyses were conducted with SAS version 9.4. Data were analyzed from July 18, 2023, to June 11, 2025.

A preplanned sensitivity analysis was conducted, in which medication discontinuation was defined as 14 nonhospitalized days without use of the medication, an alternative definition of OAT discontinuation.^15^ We conducted post hoc sensitivity analyses excluding treatment initiations that occurred between March 1, 2019, and February 28, 2021, to evaluate whether the study findings were affected by changes associated with the COVID-19 pandemic.

## Results

The cohort included 72 717 new users of buprenorphine-naloxone or methadone (45 256 [62.2%] male; median [IQR] age, 35 [28-46] years), with 34 538 individuals (47.5%) receiving methadone and 38 179 individuals (52.5%) receiving buprenorphine-naloxone (Table 1 and Table 2). The percentage of individuals starting methadone decreased from 61.7% in 2014 to 2016, to 40.6% in 2017 to 2019 and 34.7% in 2020 to 2022 (Table 1 and Table 2).

**Table 1.** Baseline Characteristics of Methadone Cohort

Characteristic	Individuals, No. (%)			
	All years (n = 34 538)	2014-2016 (n = 18 017)	2017-2019 (n = 9860)	2020-2022 (n = 6661)
Age, y				
Median (IQR)	34 (27-44)	33 (26-42)	35 (28-46)	36 (28-47)
15-24	5212 (15.1)	3036 (16.9)	1364 (13.8)	812 (12.2)
25-34	12 808 (37.1)	7082 (39.3)	3503 (35.5)	2223 (33.4)
35-44	8169 (23.7)	4117 (22.9)	2360 (23.9)	1692 (25.4)
45-54	5005 (14.5)	2555 (14.2)	1521 (15.4)	929 (13.9)
55-64	2311 (6.7)	950 (5.3)	747 (7.6)	614 (9.2)
≥65	1033 (3.0)	277 (1.5)	365 (3.7)	391 (5.9)
Sex				
Female	12 431 (36.0)	6454 (35.8)	3602 (36.5)	2375 (35.7)
Male	22 107 (64.0)	11 563 (64.2)	6258 (63.5)	4286 (64.3)
Urban/rural residence				
Urban	29 478 (85.3)	15 372 (85.3)	8432 (85.5)	5674 (85.2)
Rural	4759 (13.8)	2516 (14.0)	1345 (13.6)	898 (13.5)
Missing	301 (0.9)	129 (0.7)	83 (0.8)	89 (1.3)
Neighborhood income quintile				
1	13 075 (37.9)	6896 (38.3)	3651 (37.0)	2528 (38.0)
2	7717 (22.3)	3988 (22.1)	2274 (23.1)	1455 (21.8)
3	5759 (16.7)	2981 (16.5)	1668 (16.9)	1110 (16.7)
4	4361 (12.6)	2267 (12.6)	1236 (12.5)	858 (12.9)
5	3252 (9.4)	1707 (9.5)	929 (9.4)	616 (9.2)
Missing	374 (1.1)	178 (1.0)	102 (1.0)	94 (1.4)
Comorbidities				
HIV	216 (0.6)	133 (0.7)	46 (0.5)	37 (0.6)
Diabetes	1953 (5.7)	894 (5.0)	603 (6.1)	456 (6.8)
COPD	2980 (8.6)	1369 (7.6)	977 (9.9)	634 (9.5)
Asthma	8121 (23.5)	4221 (23.4)	2337 (23.7)	1563 (23.5)
Hypertension	3882 (11.2)	1786 (9.9)	1217 (12.3)	879 (13.2)
Opioid overdose				
In past 30 d	191 (0.6)	79 (0.4)	84 (0.9)	28 (0.4)
In past 365 d	954 (2.8)	365 (2.0)	440 (4.5)	149 (2.2)
ED visit for any mental health or addiction in past year	5869 (17.0)	2883 (16.0)	1690 (17.1)	1296 (19.5)
Johns Hopkins ADGs				
0	3552 (10.3)	1657 (9.2)	1088 (11.0)	807 (12.1)
1	4235 (12.3)	2333 (12.9)	1123 (11.4)	779 (11.7)
2	3900 (11.3)	2088 (11.6)	1088 (11.0)	724 (10.9)
3	3774 (10.9)	2005 (11.1)	1110 (11.3)	659 (9.9)
4	3432 (9.9)	1865 (10.4)	979 (9.9)	588 (8.8)
5	3174 (9.2)	1719 (9.5)	893 (9.1)	562 (8.4)
6	2663 (7.7)	1425 (7.9)	749 (7.6)	489 (7.3)
≥7	9808 (28.4)	4925 (27.3)	2830 (28.7)	2053 (30.8)

Abbreviations: ADG, Aggregated Diagnosis Groups; COPD, chronic obstructive pulmonary disease; ED, emergency department.

**Table 2.** Baseline Characteristics of Buprenorphine-Naloxone Cohort

Characteristic	Individuals, No. (%)			
	All years (n = 38 179)	2014-2016 (n = 11 183)	2017-2019 (n = 14 451)	2020-2022 (n = 12 545)
Age, y				
Median (IQR)	36 (28-49)	34 (27-45)	37 (28-50)	38 (29-51)
15-24	5360 (14.0)	1833 (16.4)	1924 (13.3)	1603 (12.8)
25-34	11 958 (31.3)	4037 (36.1)	4296 (29.7)	3625 (28.9)
35-44	8649 (22.7)	2511 (22.5)	3296 (22.8)	2842 (22.7)
45-54	6200 (16.2)	1745 (15.6)	2471 (17.1)	1984 (15.8)
55-64	4099 (10.7)	836 (7.5)	1655 (11.5)	1608 (12.8)
≥65	1913 (5.0)	221 (2.0)	809 (5.6)	883 (7.0)
Sex				
Female	15 030 (39.4)	4435 (39.7)	5763 (39.9)	4832 (38.5)
Male	23 149 (60.6)	6748 (60.3)	8688 (60.1)	7713 (61.5)
Urban/rural residence				
Urban	30 689 (80.4)	8696 (77.8)	11 719 (81.1)	10 274 (81.9)
Rural	7226 (18.9)	2429 (21.7)	2637 (18.2)	2160 (17.2)
Missing	264 (0.7)	58 (0.5)	95 (0.7)	111 (0.9)
Neighborhood income quintile				
1	13 873 (36.3)	4367 (39.1)	5217 (36.1)	4289 (34.2)
2	7925 (20.8)	2187 (19.6)	3067 (21.2)	2671 (21.3)
3	6290 (16.5)	1719 (15.4)	2416 (16.7)	2155 (17.2)
4	5184 (13.6)	1489 (13.3)	1927 (13.3)	1768 (14.1)
5	4607 (12.1)	1359 (12.2)	1707 (11.8)	1541 (12.3)
Missing	300 (0.8)	62 (0.6)	117 (0.8)	121 (1.0)
Comorbidities				
HIV	217 (0.6)	53 (0.5)	99 (0.7)	65 (0.5)
Diabetes	3417 (8.9)	725 (6.5)	1404 (9.7)	1288 (10.3)
COPD	4308 (11.3)	955 (8.5)	1845 (12.8)	1508 (12.0)
Asthma	9192 (24.1)	2450 (21.9)	3519 (24.4)	3223 (25.7)
Hypertension	6496 (17.0)	1466 (13.1)	2646 (18.3)	2384 (19.0)
Opioid overdose				
In past 30 d	250 (0.7)	58 (0.5)	145 (1.0)	47 (0.4)
In past 365 d	917 (2.4)	244 (2.2)	472 (3.3)	201 (1.6)
ED visit for any mental health or addiction in past year	8247 (21.6)	2081 (18.6)	3160 (21.9)	3006 (24.0)
Johns Hopkins ADGs				
0	2427 (6.4)	716 (6.4)	858 (5.9)	853 (6.8)
1	3589 (9.4)	1230 (11.0)	1219 (8.4)	1140 (9.1)
2	3634 (9.5)	1157 (10.3)	1382 (9.6)	1095 (8.7)
3	3916 (10.3)	1257 (11.2)	1422 (9.8)	1237 (9.9)
4	3815 (10.0)	1179 (10.5)	1420 (9.8)	1216 (9.7)
5	3747 (9.8)	1130 (10.1)	1419 (9.8)	1198 (9.5)
6	3428 (9.0)	962 (8.6)	1340 (9.3)	1126 (9.0)
≥7	13 623 (35.7)	3552 (31.8)	5391 (37.3)	4680 (37.3)

Abbreviations: ADG, Aggregated Diagnosis Group; COPD, chronic obstructive pulmonary disease; ED, emergency department.

### Methadone

During 2014 to 2022, 34 538 individuals (mean [SD] age, 36.6 [12.6] years) met the inclusion criteria for a new initiation of methadone. Of these new initiations, 18 017 (52.2%) occurred during 2014 to 2016, 9860 (28.5%) occurred during 2017 to 2019, and 6661 (19.3%) occurred during 2020 to 2022. Comorbidities and sociodemographic characteristics were similar among individuals starting methadone during the 3 time periods (Table 1). A total of 954 individuals (2.8%) had an ED visit for and opioid-related overdose over the year prior to initiation.

**Figure 1.**
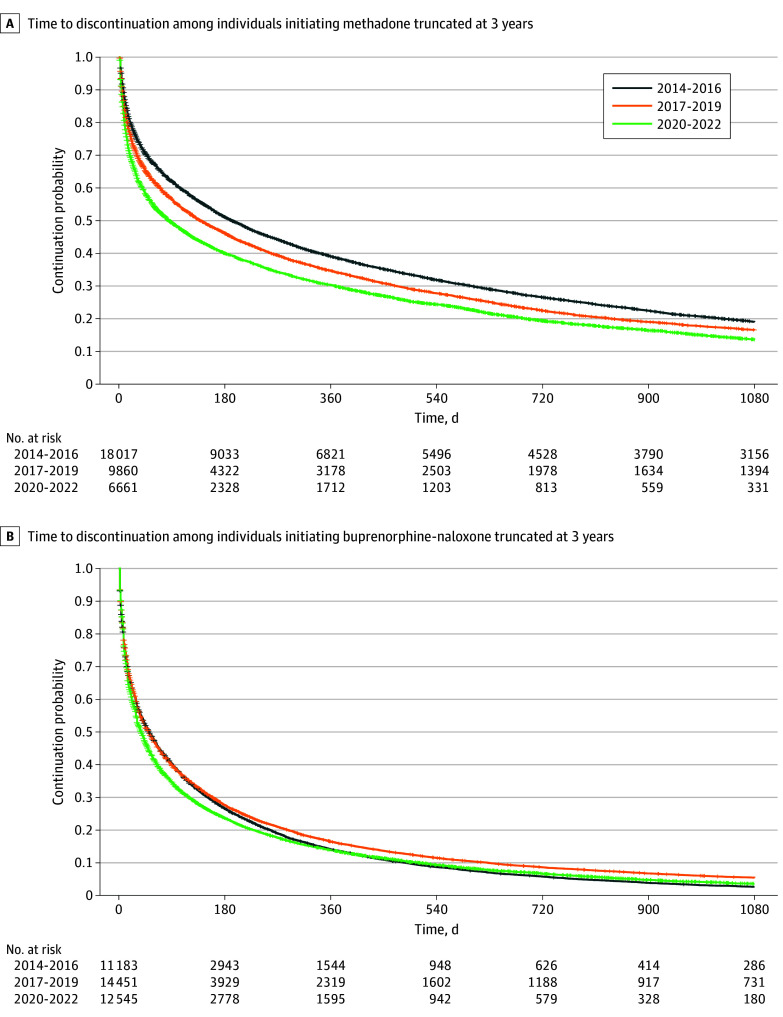
Kaplan-Meier Curves for Time to Discontinuation Among Individuals Initiating Methadone or Buprenorphine-Naloxone Kaplan-Meier curves were truncated at 3 years for visualization. Full Kaplan-Meier curves are presented in eFigure 1 and eFigure 3 in Supplement 1.

**Figure 2.**
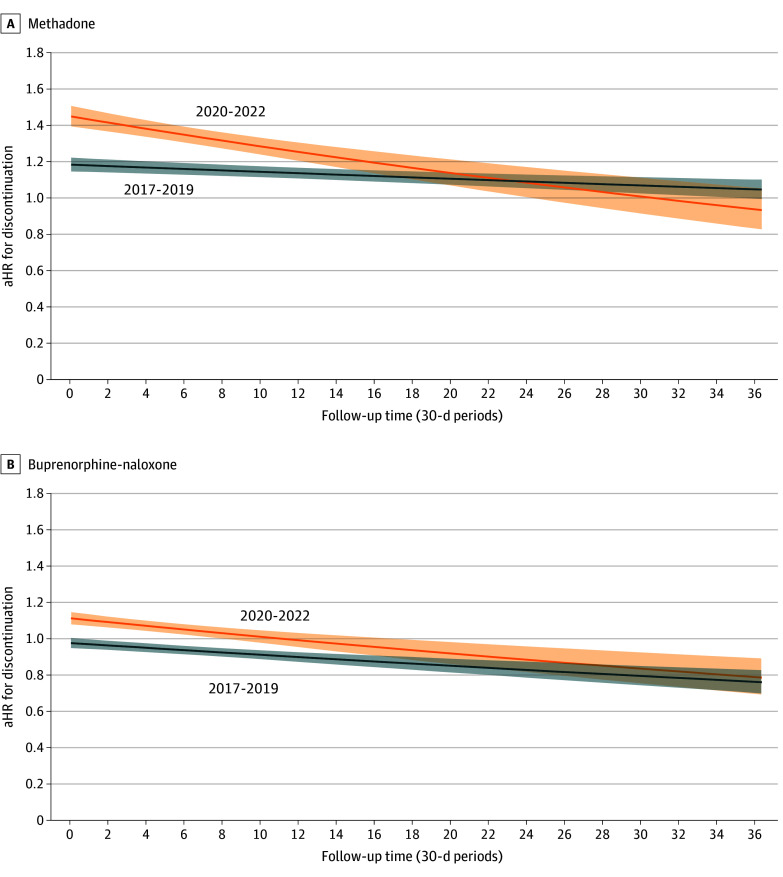
Adjusted Hazard Ratios (aHRs) for Time to Discontinuation as a Function of Time for Methadone and Buprenorphine-Naloxone For all analyses, the reference period was 2014 to 2016.

Among individuals starting methadone, median treatment duration decreased from 193 (95% CI, 185-202) days in 2014 to 2016 to 139 (95% CI, 130-149) days in 2017 to 2019 and 86 (95% CI,78-95) days in 2020 to 2022 (Figure 1 and Table 3). Compared with individuals starting methadone during the 2014 to 2016 reference period, individuals starting methadone during 2017 to 2019 and 2020 to 2022 had a significantly higher hazard for discontinuation in the unadjusted model (Table 3; eFigure 2 in Supplement 1). In the adjusted model, there were significant negative interactions between the initiation period and time taking methadone, such that relative hazards for discontinuation were highest at treatment initiation and decreased thereafter (Figure 2). In adjusted models and compared with individuals who initiated methadone treatment in 2014 to 2016, there was greater hazard for treatment discontinuation for individuals who initiated methadone treatment during 2017 to 2019 (adjusted hazard ratio [aHR], 1.18 [95% CI, 1.15-1.22]; *P* < .001) or 2020 to 2022 (aHR, 1.45 [95% CI, 1.39-1.51]; *P* < .001).

**Table 3.** Median Treatment Duration and Hazard Ratios for Treatment Discontinuation by Period of Medication Initiation

Period	Treatment duration, median (95% CI), d	Unadjusted HR (95% CI)	*P* value	Adjusted HR (95% CI)^a^	*P* value
**Methadone**					
2014-2016	193 (185-202)	1 [Reference]	NA	1 [Reference]	NA
2017-2019	139 (130-149)	1.11 (1.08-1.14)	<.001	1.18 (1.15-1.22)	<.001
2020-2022	86 (78-95)	1.29 (1.25-1.34)	<.001	1.45 (1.39-1.51)	<.001
**Buprenorphine-naloxone**					
2014-2016	51 (49-54)	1 [Reference]	NA	1 [Reference]	NA
2017-2019	50 (48-53)	0.93 (0.90-0.95)	<.001	0.98 (0.95-1.00)	.09
2020-2022	38 (36-40)	1.04 (1.02-1.07)	.002	1.11 (1.08-1.15)	<.001

Abbreviations: HR, hazard ratio; NA, not applicable.

^a^ In each adjusted model, there was a significant interaction between index period and time since index. HRs for discontinuation gradually decreased with time elapsed after the index date.

Age categories were significantly associated with methadone discontinuation in adjusted models (eTable 2 in Supplement 1). Compared with individuals ages 35 to 44 years, individuals ages 15 to 24 years (aHR, 1.32 [95% CI, 1.27-1.37]), 25 to 34 years (aHR, 1.17 [95% CI, 1.14-1.21]) and 65 years or older (aHR, 1.27 [95% CI, 1.16-1.38) had increased hazards for discontinuation, while individuals ages 45 to 54 (aHR, 0.88 [95% CI, 0.85-0.91]) and 55 to 64 years (aHR, 0.78 [95% CI, 0.74-0.82]) had decreased hazards for discontinuation (eTable 2 in Supplement 1). The HRs for additional covariates used in model adjustment are shown in eTable 2 in Supplement 1. Rurality, increased number of Johns Hopkins ADGs, and decreased neighborhood income quintile were associated with an increased hazard of discontinuation. Censoring ranged from 8.4% in 2014 to 2016 to 23.5% in 2020 to 2022; reasons for censoring are presented in eTable 3 in Supplement 1.

### Buprenorphine-Naloxone

During 2014 to 2022, 38 179 individuals (mean [SD] age, 39.0 [13.9] years) had new initiations of buprenorphine-naloxone meeting cohort inclusion criteria. Of these new initiations, 11 183 (29.3%) occurred during 2014 to 2016, 14 451 (37.9%) occurred during 2017 to 2019, and 12 545 (32.9%) occurred during 2020 to 2022. Comorbidities and sociodemographic characteristics were similar among individuals starting buprenorphine-naloxone during the 3 time periods (Table 2). A total of 917 individuals (2.4%) had an ED visit for an opioid-related overdose in the year prior to initiation.

Among individuals starting buprenorphine-naloxone, median treatment duration decreased from 51 (95% CI, 49-54) days in 2014 to 2016 and 50 (95% CI, 48-53) days in 2017 to 2019 to 38 (95% CI, 36-40) days in 2020 to 2022 (Figure 2 and Table 3; eFigure 3 in Supplement 1). Compared with individuals starting buprenorphine-naloxone during the 2014 to 2016 reference period, individuals starting buprenorphine-naloxone during 2020 to 2022 had a significantly higher hazard of discontinuation in both unadjusted and adjusted (aHR, 1.11 [95% CI, 1.08-1.15]; *P* < .001) models (Table 3; eFigure 4 in Supplement 1). The hazard for discontinuation for individuals who initiated buprenorphine-naloxone in 2017 to 2019 was not significantly different from individuals who initiated in 2014 to 2016 (aHR, 0.98 [95% CI, 0.95-1.00]; *P* = .09). There were statistically significant, yet small time × index period interactions, such that HRs decreased with time after treatment initiation (Figure 2**)**.

The HRs for additional covariates used in model adjustment are in eTable 4 in Supplement 1. Age categories were significantly associated with buprenorphine-naloxone discontinuation. Compared with individuals ages 35 to 44 years, individuals ages 15 to 24 years (aHR, 1.42 [95% CI, 1.38-1.47]), 25 to 34 years (aHR, 1.18 [95% CI, 1.15-1.21]) and 65 years or older (aHR, 1.07 [95% CI, 1.02-1.13]) had increased hazards for discontinuation, while individuals ages 45 to 54 years (aHR, 0.94 [95% CI, 0.91-0.97]) and 55 to 64 years (aHR, 0.91 [95% CI, 0.88-0.95]) had decreased hazards for discontinuation (eTable 4 in Supplement 1). Rurality, increased number of Johns Hopkins ADGs, male sex, and decreased neighborhood income quintile were associated with increased hazards of discontinuation. Censoring ranged from 1.5% in 2014 to 2016 to 8.2% in 2020 to 2022; reasons for censoring are presented in eTable 5 in Supplement 1).

### Sensitivity Analyses

In the preplanned sensitivity analyses redefining the outcome as 14 days without use of the OAT dispensed on the index date, the associations between period of initiation and treatment duration were similar, although treatment durations were longer across all medications and time-periods (eTable 6 and eTable 7 in Supplement 1). Among individuals initiating methadone, the median treatment durations were 370 (95% CI, 354-387) days during 2014 to 2016, 246 (95% CI, 230-262) days during 2017 to 2019, and 152 (95% CI, 137-166) days during 2020 to 2022. Among individuals initiating buprenorphine-naloxone, the median treatment durations were 94 (95% CI, 88-100) days during 2014 to 2016, 95 (95% CI, 90-101) days during 2017 to 2019, and 64 (95% CI, 61-67) days during 2020 to 2022. In the second sensitivity analysis in which all new initiations during the period from March 2019 to February 2021 were excluded, treatment durations and associations remained similar to the primary analysis (eTables 8-10 in Supplement 1).

## Discussion

This retrospective cohort study characterizes changes in treatment duration for new initiations of methadone and buprenorphine-naloxone in Ontario during a period of increasing fentanyl prevalence in the illicit opioid supply. Treatment duration for individuals starting methadone decreased significantly between 2014 to 2016 and 2020 to 2022. By 2020 to 2022, the median treatment duration for individuals starting methadone had decreased by more than half, corresponding to a median treatment duration period that was approximately 3 months shorter. Among individuals starting buprenorphine-naloxone, treatment duration was also statistically significantly shorter during 2020 to 2022 than during 2014 to 2016, although the decrease in treatment duration was less prominent than with methadone. There were also important associations between age and hazards of methadone or buprenorphine-naloxone discontinuation, with youths ages 15 to 24 years having the highest hazards of medication discontinuation.

The decreases in methadone and buprenorphine-naloxone treatment duration are consistent with patient reports and clinician experiences that standard OAT paradigms have been less effective in providing relief from withdrawal symptoms and cravings among individuals using fentanyl.^4,6,16,17,18^ Studies from the US evaluating urine drug screens have found that many patients continue to have exposure to fentanyl after starting methadone.^19,20,21^ These findings are also consistent with findings from the US that rates of before–medically advised discharges among patients with OUD admitted to the hospital have increased as fentanyl has spread in the US.^22,23^ Treatment duration is an important proxy of treatment effectiveness and has been included as a core outcome within the US National Institute on Drug Abuse Clinical Trials Network core outcome set for evaluation of OUD treatment.^7^

It is unclear from our data why the decreases in treatment duration were more prominent with methadone than with buprenorphine-naloxone. Although we are not aware of previous population-based studies that evaluated methadone treatment duration, a large US study has evaluated buprenorphine treatment retention.^24^ Using claims from an all-payer database capturing 92% of retail pharmacy dispensations, Chua et al^24^ found that 180-day buprenorphine treatment retention was minimally changed between 2016 and 2022. Several factors may explain the different buprenorphine results between our study and the study by Chua et al,^24^ including differences in jurisdictions, end points (180-day retention vs time to discontinuation), and definitions of treatment retention (a single monthly buprenorphine fill was required in the prior study), and the population-based nature of our study. Further studies are particularly needed to evaluate our findings about methadone treatment duration in other jurisdictions.

Although our study found decreasing methadone and buprenorphine-naloxone treatment duration in Ontario during a period coinciding with the spread of fentanyl, this study does not establish a causal link between the changing illicit opioid supply and decreasing treatment duration. Patient decisions to discontinue methadone or buprenorphine-naloxone are multifactorial, including both intrinsic factors (eg, concerns about adverse effects, desire for opioid-free recovery, desire for opioid use) and extrinsic factors (eg, stigma and health system–based barriers).^25,26^ The illicit opioid supply in Ontario has undergone other changes beyond the spread of fentanyl, including the spread of benzodiazepines admixed into the opioid supply.^27^ The decrease in treatment duration may be mediated through other health system changes, although these potential mechanisms are less consistent with the available data. For example, the treatment delivery system for methadone in Ontario underwent changes to reduce barriers and improve access.^28^ In 2018, the Canadian federal government removed the requirement that physicians obtain an exemption from the federal Controlled Substances Act to prescribe methadone, and in 2021, Ontario’s physician regulatory body removed special registration and training requirements for prescribing methadone.^28^ Additionally, a network of clinics providing low-threshold, same-day initiation of OAT has developed in the province, improving accessibility of methadone.^29^ The 2014 to 2022 period also featured changing prescribing practices, in which the proportion of individuals initiating OAT who received methadone decreased, with a corresponding increase in the proportion of individuals receiving buprenorphine-naloxone. However, measures of comorbidity and sociodemographic characteristics of individuals receiving the medications remained similar across time periods, reducing the likelihood of differential access or prescribing practices being responsible for the results. Furthermore, data from low-threshold clinics in Ontario suggest that patients starting OAT in these settings have similar or better outcomes to patients starting the medication in other settings.^29^

To our knowledge, this is the first population-level study to longitudinally evaluate OUD treatment effectiveness among incident methadone and buprenorphine-naloxone recipients during the spread of fentanyl.^8,15^ The results of this study have important implications for OUD treatment. First, this study highlights the importance of continuous monitoring of OUD treatment effectiveness as the illicit opioid supply and substance consumption patterns change. Second, this study supports initiatives to update protocols for providing OAT in the fentanyl era, such as providing higher doses of methadone and buprenorphine-naloxone.^6,30,31,32,33^ Third, these results highlight the importance of research into developing alternative pharmacologic treatments for OUD, beyond methadone and sublingual buprenorphine-naloxone. Newer long-acting injectable formulations of buprenorphine may have a role in promoting treatment retention and increasing medication effectiveness.^34^ Safer opioid supply and risk mitigation provision of opioids may also have a role in reducing harms associated with fentanyl use.^35^ Fifth, the decreased treatment duration with OAT supports re-evaluating whether the risks and benefits of system-based barriers, such as requirements for witnessed dosing of OAT, have changed with the spread of fentanyl, and whether these should be relaxed.^36^ Finally, this study highlights the need to improve treatment effectiveness among youth starting methadone and buprenorphine-naloxone.

This study has several strengths. The inclusion of individuals exposed to a contemporary opioid supply and the ability to compare outcomes across different time periods provides updated data to inform clinical practice and policy. The use of routinely collected administrative health data and the resulting population-based cohorts allows for high generalizability of the study results. Methodologically, this study accounts for hospitalization days, improving on previous studies that did not account for hospitalization in determining treatment duration from outpatient medication dispensing records.^15^ Additionally, this study uses a timeframe of methadone and buprenorphine-naloxone discontinuation of 5 days without medication availability, which aligns with local guidance about OAT dosing.^6,37^ Study findings remained robust with sensitivity analyses.

### Limitations

This study has some limitations. Although this study describes the reduction in treatment duration, this retrospective cohort study does not establish a causal effect between the spread of fentanyl and decreased treatment duration. Unmeasured factors affecting both OAT selection and treatment duration may confound these results. The NMS does not capture OAT dispensed in hospitals, carceral settings, or long-term care homes, and individuals may have received OAT in these settings prior to receipt of OAT in outpatient settings. The ICES databases collect data received about health care services received only in Ontario; patients may have received OAT in other jurisdictions prior to entering the cohort. Additionally, Indigenous individuals in Ontario (approximately 3% of the population^38^) who receive Federally Non-Insured Health Benefits may have misclassification of exposure and outcome due to the potential that not all OAT dispensations are linked to an Ontario Health Insurance Plan–based ICES Key Number.

## Conclusions

This cohort study found that treatment duration among individuals starting methadone or buprenorphine-naloxone in Ontario, Canada, during 2020 to 2022 was lower than in 2014 to 2016, coinciding with the spread of fentanyl into the opioid supply. This study highlights the importance of ongoing monitoring of OAT treatment effectiveness as the opioid crisis evolves. The results of this study suggest that further research is needed to improve treatment retention and provide effective OUD treatment.

## Notes

Supplement 1. **eTable 1.** ICES databases and definitions used in analysis **eTable 2.** Survival models and model diagnostics for adjusted models in primary analysis of methadone treatment duration **eTable 3.** Survival models and model diagnostics for adjusted models primary analysis of buprenorphine treatment duration **eFigure 1.** Kaplan-Meier curves for time to discontinuation among individuals initiating methadone **eFigure 2.** Log-log plot for adjusted methadone survival model with five-day discontinuation outcome **eTable 4.** Cox proportional hazard models and model diagnostics for primary analysis of buprenorphine-naloxone treatment duration **eTable 5.** Reasons for discontinuation and/or censoring among individuals receiving buprenorphine-naloxone **eFigure 3.** Kaplan-Meier curves for time to discontinuation among individuals initiating buprenorphine-naloxone **eFigure 4.** Log-log plot for adjusted buprenorphine-naloxone survival model with five-day discontinuation outcome **eTable 6.** Multivariable Cox proportional hazard regression for methadone treatment discontinuation in sensitivity analysis with 14 day period for discontinuation **eTable 7.** Multivariable Cox proportional hazard regression for buprenorphine-naloxone treatment discontinuation in sensitivity analysis with 14 day period for discontinuation **eTable 8.** Median treatment durations for methadone and buprenorphine in sensitivity analysis excluding initiations between March 1, 2019 and February 28, 2021 **eTable 9.** Multivariable Cox proportional hazard regression for methadone treatment discontinuation in sensitivity analysis excluding initiations between March 1, 2019 and February 28, 2021 **eTable 10.** Multivariable Cox proportional hazard regression for buprenorphine-naloxone treatment discontinuation in sensitivity analysis excluding initiations between March 1, 2019 and February 28, 2021

Supplement 2.
Data Sharing Statement

## References

[1] Jones NR, Hickman M, Nielsen S, . The impact of opioid agonist treatment on fatal and non-fatal drug overdose among people with a history of opioid dependence in NSW, Australia, 2001-2018: Findings from the OATS retrospective linkage study. Drug Alcohol Depend. 2022;236:109464. doi:10.1016/j.drugalcdep.2022.109464 35523111

[2] American Society of Addiction Medicine (ASAM). ASAM National Practice Guideline for the Treatment of Opioid Use Disorder: 2020 Focused Update. ASAM; 2020.

[3] Sordo L, Barrio G, Bravo MJ, . Mortality risk during and after opioid substitution treatment: systematic review and meta-analysis of cohort studies. BMJ. 2017;357:j1550. doi:10.1136/bmj.j1550 28446428 PMC5421454

[4] Thakrar AP, Kleinman RA. Opioid withdrawal management in the fentanyl era. Addiction. 2022;117(9):2560-2561. doi:10.1111/add.15893 35373864

[5] Kleinman RA. Fentanyl, carfentanil and other fentanyl analogues in Canada’s illicit opioid supply: A cross-sectional study. Drug Alcohol Depend Rep. Published online May 23, 2024. doi:10.1016/j.dadr.2024.100240 39035468 PMC11259693

[6] Bromley L, Kahan M, Regenstreif L, Srivastava A, Wyman J. Methadone treatment for people who use fentanyl: recommendations. Accessed March 12, 2024. https://www.metaphi.ca/wp-content/uploads/Guide_MethadoneForFentanyl.pdf

[7] Karnik NS, Marsden J, McCluskey C, . The opioid use disorder core outcomes set (OUD-COS) for treatment research: findings from a Delphi consensus study. Addiction. 2022;117(9):2438-2447. doi:10.1111/add.15875 35293064 PMC9543602

[8] Pearce LA, Min JE, Piske M, . Opioid agonist treatment and risk of mortality during opioid overdose public health emergency: population based retrospective cohort study. BMJ. 2020;368:m772. doi:10.1136/bmj.m772 32234712 PMC7190018

[9] Langan SM, Schmidt SA, Wing K, . The reporting of studies conducted using observational routinely collected health data statement for pharmacoepidemiology (RECORD-PE). BMJ. 2018;363:k3532. doi:10.1136/bmj.k3532 30429167 PMC6234471

[10] Ministry of Health, Government of Ontario. Ontario Drug Programs Reference Manual. King’s Printer for Ontario; 2023.

[11] Public Health Ontario. Interactive Opioid Tool. Accessed June 7, 2025. https://www.publichealthontario.ca/en/Data-and-Analysis/Substance-Use/Interactive-Opioid-Tool#/

[12] Stafford C, Marrero WJ, Naumann RB, Lich KH, Wakeman S, Jalali MS. Identifying key risk factors for premature discontinuation of opioid use disorder treatment in the United States: a predictive modeling study. Drug Alcohol Depend. 2022;237:109507. doi:10.1016/j.drugalcdep.2022.109507 35660221

[13] Bharat C, Larney S, Barbieri S, . The effect of person, treatment and prescriber characteristics on retention in opioid agonist treatment: a 15-year retrospective cohort study. Addiction. 2021;116(11):3139-3152. doi:10.1111/add.15514 33979008

[14] Elnagdi A, McCormack D, Bozinoff N, . Opioid agonist treatment retention among people initiating methadone and buprenorphine across diverse demographic and geographic subgroups in Ontario: a population-based retrospective cohort study. Can J Addict. 2023;14(4):44. doi:10.1097/CXA.0000000000000192

[15] Gomes T, McCormack D, Bozinoff N, . Duration of use and outcomes among people with opioid use disorder initiating methadone and buprenorphine in Ontario: a population-based propensity-score matched cohort study. Addiction. 2022;117(7):1972-1981. doi:10.1111/add.15862 35257434 PMC9313829

[16] Kleinman RA, Thakrar AP. Using short-acting opioids to relieve opioid withdrawal in hospital. CMAJ. 2023;195(49):E1718-E1720. doi:10.1503/cmaj.230968 38110216 PMC10727795

[17] Knopf A. Do patients addicted to fentanyl need higher doses of methadone or buprenorphine? Alcohol Drug Abuse Week. 2022;34(32):1-7. doi:10.1002/adaw.33525

[18] Buresh M, Nahvi S, Steiger S, Weinstein ZM. Adapting methadone inductions to the fentanyl era. J Subst Abuse Treat. 2022;141:108832. doi:10.1016/j.jsat.2022.108832 35870437

[19] Arfken CL, Suchanek J, Greenwald MK. Characterizing fentanyl use in methadone-maintained clients. J Subst Abuse Treat. 2017;75:17-21. doi:10.1016/j.jsat.2017.01.004 28237050

[20] Stone AC, Carroll JJ, Rich JD, Green TC. One year of methadone maintenance treatment in a fentanyl endemic area: safety, repeated exposure, retention, and remission. J Subst Abuse Treat. 2020;115:108031. doi:10.1016/j.jsat.2020.108031 32600619 PMC10347815

[21] Saloner B, Whitley P, Dawson E, Passik S, Gordon AJ, Stein BD. Polydrug use among patients on methadone medication treatment: evidence from urine drug testing to inform patient safety. Addiction. 2023;118(8):1549-1556. doi:10.1111/add.16180 37158468 PMC10330099

[22] Thakrar AP, Lowenstein M, Greysen SR, Delgado MK. Trends in before medically advised discharges for patients with opioid use disorder, 2016-2020. JAMA. 2023;330(23):2302-2304. doi:10.1001/jama.2023.21288 38048121 PMC10696509

[23] Kleinman RA, Brothers TD, Morris NP. Retiring the “against medical advice” discharge. Ann Intern Med. 2022;175(12):1761-1762. doi:10.7326/M22-2964 36442060

[24] Chua KP, Nguyen TD, Zhang J, Conti RM, Lagisetty P, Bohnert AS. Trends in buprenorphine initiation and retention in the United States, 2016-2022. JAMA. 2023;329(16):1402-1404. doi:10.1001/jama.2023.1207 37097363 PMC10130945

[25] Thakrar AP, Pytell JD, Stoller KB, Walters V, Weiss RD, Chander G. Transitioning off methadone: a qualitative study exploring why patients discontinue methadone treatment for opioid use disorder. J Subst Use Addict Treat. 2023;150:209055. doi:10.1016/j.josat.2023.209055 37088398 PMC10330232

[26] Wyse JJ, Eckhardt A, Waller D, . Patients’ perspectives on discontinuing buprenorphine for the treatment of opioid use disorder. J Addict Med. 2024;18(3):300-305. doi:10.1097/ADM.0000000000001292 38498620 PMC11853618

[27] Pardo B. Insights into mixing fentanyl and benzodiazepines from Canadian drug seizures. JAMA Psychiatry. 2022;79(1):81-83. doi:10.1001/jamapsychiatry.2021.3292 34787646 PMC8600450

[28] Kleinman RA, Brothers TD, Danilewitz M, Bahji A. Office-based methadone prescribing for opioid use disorder: the Canadian model. J Addict Med. 2022;16(5):499-504. doi:10.1097/ADM.0000000000000950 35020695 PMC9271524

[29] Corace K, Thavorn K, Suschinsky K, . Rapid access addiction medicine clinics for people with problematic opioid use. JAMA Netw Open. 2023;6(11):e2344528. doi:10.1001/jamanetworkopen.2023.44528 37991762 PMC10665968

[30] Weimer MB, Herring AA, Kawasaki SS, Meyer M, Kleykamp BA, Ramsey KS. ASAM clinical considerations: buprenorphine treatment of opioid use disorder for individuals using high-potency synthetic opioids. J Addict Med. 2023;17(6):632-639. doi:10.1097/ADM.0000000000001202 37934520

[31] Wong S, Fabiano N, Webber D, Kleinman RA. High-dose buprenorphine initiation: a scoping review. J Addict Med. 2024;18(4):349-359. doi:10.1097/ADM.0000000000001296 38757944

[32] Kleinman RA, Wakeman SE. Treating opioid withdrawal in the hospital: a role for short-acting opioids. Ann Intern Med. 2022;175(2):283-284. doi:10.7326/M21-3968 34807718

[33] Fiellin DA. Buprenorphine initiation in the era of high-potency synthetic opioids: a call for community-based participatory research to help learning health systems provide precision medicine for opioid use disorder. J Addict Med. 2022;16(6):e348-e349. doi:10.1097/ADM.0000000000001007 36166675

[34] Iacono A, Wang T, Tadrous M, . Characteristics, treatment patterns and retention with extended-release subcutaneous buprenorphine for opioid use disorder: a population-based cohort study in Ontario, Canada. Drug Alcohol Depend. 2024;254:111032. doi:10.1016/j.drugalcdep.2023.111032 38043224

[35] Slaunwhite A, Min JE, Palis H, . Effect of risk mitigation guidance opioid and stimulant dispensations on mortality and acute care visits during dual public health emergencies: retrospective cohort study. BMJ. 2024;384:e076336. doi:10.1136/bmj-2023-076336 38199614 PMC10777271

[36] Kleinman RA, Nielsen S, Weiss RD. Is daily supervised buprenorphine-naloxone dosing necessary? BMJ. 2022;378:e071467. doi:10.1136/bmj-2022-071467 35973725

[37] College of Physicians and Surgeons of Ontario. Methadone Maintenance Treatment: Program Standards and Clinical Guidelines. College of Physicians and Surgeons of Ontario; 2011.

[38] Indigenous population continues to grow and is much younger than the non-Indigenous population, although the pace of growth has slowed. News release. Statistics Canada. September 21, 2022. Accessed July 10, 2024. https://www150.statcan.gc.ca/n1/daily-quotidien/220921/dq220921a-eng.htm

