# Supplementary material for: Duration of Methadone and Buprenorphine-Naloxone Treatment
Source: JAMA Netw Open. 2025 Jul 1;8(7):e2518389. doi: 10.1001/jamanetworkopen.2025.18389 (PMC12215571; doi:10.1001/jamanetworkopen.2025.18389)
Supplement: Supplement 1. — eTable 1. ICES databases and definitions used in analysis eTable 2. Survival models and model diagnostics for adjusted models in primary analysis of methadone treatment duration eTable 3. Survival models and model diagnostics for adjusted models primary analysis of buprenorphine treatment duration eFigure 1. Kaplan-Meier curves for time to discontinuation among individuals initiating methadone eFigure 2. Log-log plot for adjusted methadone survival model with five-day discontinuation outcome eTable 4. Cox proportional hazard models and model diagnostics for primary analysis of buprenorphine-naloxone treatment duration eTable 5. Reasons for discontinuation and/or censoring among individuals receiving buprenorphine-naloxone eFigure 3. Kaplan-Meier curves for time to discontinuation among individuals initiating buprenorphine-naloxone eFigure 4. Log-log plot for adjusted buprenorphine-naloxone survival model with five-day discontinuation outcome eTable 6. Multivariable Cox proportional hazard regression for methadone treatment discontinuation in sensitivity analysis with 14 day period for discontinuation eTable 7. Multivariable Cox proportional hazard regression for buprenorphine-naloxone treatment discontinuation in sensitivity analysis with 14 day period for discontinuation eTable 8. Median treatment durations for methadone and buprenorphine in sensitivity analysis excluding initiations between March 1, 2019 and February 28, 2021 eTable 9. Multivariable Cox proportional hazard regression for methadone treatment discontinuation in sensitivity analysis excluding initiations between March 1, 2019 and February 28, 2021 eTable 10. Multivariable Cox proportional hazard regression for buprenorphine-naloxone treatment discontinuation in sensitivity analysis excluding initiations between March 1, 2019 and February 28, 2021 [file jamanetwopen-e2518389-s001.pdf]

Supplemental Online Content

Kleinman RA, Kurdyak P. Duration of methadone and buprenorphine-naloxone treatment. *JAMA Netw Open*. 2025;8(7):e2518389. doi:10.1001/jamanetworkopen.2025.18389

eTable 1. ICES databases and definitions used in analysis
eTable 2. Survival models and model diagnostics for adjusted models in primary analysis of methadone treatment duration
eTable 3. Survival models and model diagnostics for adjusted models primary analysis of buprenorphine treatment duration
eFigure 1. Kaplan-Meier curves for time to discontinuation among individuals initiating methadone
eFigure 2. Log-log plot for adjusted methadone survival model with five-day discontinuation outcome
eTable 4. Cox proportional hazard models and model diagnostics for primary analysis of buprenorphine-naloxone treatment duration
eTable 5. Reasons for discontinuation and/or censoring among individuals receiving buprenorphine-naloxone
eFigure 3. Kaplan-Meier curves for time to discontinuation among individuals initiating buprenorphine-naloxone
eFigure 4. Log-log plot for adjusted buprenorphine-naloxone survival model with five-day discontinuation outcome
eTable 6. Multivariable Cox proportional hazard regression for methadone treatment discontinuation in sensitivity analysis with 14 day period for discontinuation
eTable 7. Multivariable Cox proportional hazard regression for buprenorphine-naloxone treatment discontinuation in sensitivity analysis with 14 day period for discontinuation
eTable 8. Median treatment durations for methadone and buprenorphine in sensitivity analysis excluding initiations between March 1, 2019 and February 28, 2021
eTable 9. Multivariable Cox proportional hazard regression for methadone treatment discontinuation in sensitivity analysis excluding initiations between March 1, 2019 and February 28, 2021
eTable 10. Multivariable Cox proportional hazard regression for buprenorphine-naloxone treatment discontinuation in sensitivity analysis excluding initiations between March 1, 2019 and February 28, 2021

This supplemental material has been provided by the authors to give readers additional information about their work.

© 2025 Kleinman RA et al. *JAMA Network Open*.

ICES Databases and Definitions Used in Analysis

**Exposure/outcome/covariate defined in analysis**	**ICES Database**
Exposure to opioid agonist treatment*	Narcotic Monitoring System
Opioid agonist treatment discontinuation	Narcotic Monitoring System
Hospitalization days	Discharge Abstract Database
Non-fatal opioid overdose**	National Ambulatory Care Reporting System
Asthma	Ontario Asthma Dataset
Chronic obstructive pulmonary disease	Chronic Obstructive Pulmonary Disease Dataset
Rurality, neighbourhood income quintile, death, loss of Ontario Health Insurance Program coverage	Registered Person Database
Human immunodeficiency virus	Ontario HIV Database
Hypertension	Ontario Hypertension Dataset
Johns Hopkins Aggregated Diagnosis Groups (ADGs)	National Ambulatory Care Reporting System<br>Discharge Abstract Database<br>Ontario Health Insurance Plan Claims Database<br>Same Day Surgery Database

eTable 1. ICES Databases and Definitions Used in Analysis.

*Opioid agonist treatments were identified using Drug Identification Numbers/Product Identification Numbers:

<u>Sublingual buprenorphine-naloxone</u>

2408090, 2408104, 2424851, 2424878, 2295695, 2295709, 2468085, 2468093, 2502313, 2502321, 2502348, 2502356, 2453908, 2453916

<u>Liquid Methadone</u>

02481979, 02394596, 02394618, 02495872, 02495880, 2244290, 09850619, 09857499, 02495783

<u>Slow-release oral morphine</u>

2184435, 2184443, 2184451, 2242163, 2019930, 2019949, 2019957, 2019965, 2177749, 2177757

<u>Buprenorphine extended-release</u>

Probuphine: 02474921

Sublocade: 02483084, 02483092, 09858127, 09858128

**Opioid overdoses were defined as unscheduled Emergency Department Visit in the National Ambulatory Care Reporting System with diagnostic codes T40.0, T40.1, T40.2, T40.3, T40.4 or T40.6 as a primary or secondary diagnosis.

© 2025 Kleinman RA et al. *JAMA Network Open*.

Cox Proportional Hazard Models and Model Diagnostics for primary analysis of methadone treatment duration

Covariate	Reference level	Hazard Ratio	P-value
Index period			
2017 – 2019	2014 – 2016	1.18 (1.15-1.22)	<.001
2020 – 2022	2014 – 2016	1.45 (1.39-1.51)	<.001
Time (30 day intervals) by index period interaction			
2017 – 2019		1.00 (0.99-1.00)	<.001
2020 – 2022		0.99 (0.98-0.99)	<.001
Age at index			
15 – 24	35 – 44	1.32 (1.27 – 1.37)	<.001
25 – 34	35 – 44	1.17 (1.14 – 1.21)	<.001
45 – 54	35 – 44	0.88 (0.85 – 0.91)	<.001
55 – 64	35 – 44	0.78 (0.74 – 0.82)	<.001
65+	35 – 44	1.27 (1.16 – 1.38)	<.001
Sex			
Male	Female	1.10 (1.07-1.12)	<.001
Rurality			
Rural	Urban	1.06 (1.02-1.09)	<.001
Missing	Urban	2.28 (1.72-3.03)	<.001
Neighbourhood income quintile			
1	5	1.08 (1.03-1.12)	<.001
2	5	1.05 (1.01-1.10)	0.02
3	5	1.02 (0.97-1.07)	0.4
4	5	0.98 (0.94-1.03)	0.5
Missing	5	0.73 (0.56-0.95)	0.02
Johns Hopkins ADGs			
1	0	1.03 (0.98-1.08)	0.2
2	0	1.02 (0.97-1.07)	0.4
3	0	1.06 (1.01-1.11)	0.03
4	0	1.06 (1.00-1.11)	0.03
5	0	1.12 (1.07-1.18)	<.001
6	0	1.13 (1.07-1.19)	<.001
7+	0	1.19 (1.14-1.24)	<.001

eTable 2. Multivariable Cox proportional hazard regression for methadone treatment discontinuation. Treatment discontinuation is operationalized as five outpatient days without dispensation of methadone or previously dispensed take-home doses covering the day.

© 2025 Kleinman RA et al. *JAMA Network Open*.

Event/censoring	Total	2014-2016	2017-2019	2020-2022
Methadone Discontinuation	30,134 (87.2%)	16,508 (91.6%)	8,532 (86.5%)	5,094 (76.5%)
Censoring				
End of follow-up (December 31, 2023)	1,926 (5.6%)	582 (3.2%)	570 (5.8%)	774 (11.6%)
Initiation of buprenorphine-XR	*1 - 5	*1 - 5	0 (0.0%)	*1 - 5
Initiation of buprenorphine-naloxone	1,099 (3.2%)	529 (2.9%)	350 (3.5%)	220 (3.3%)
Initiation of slow-release oral morphine	*418 - 422	86 (0.5%)	*75 - 79	257 (3.9%)
Death	943 (2.7%)	304 (1.7%)	328 (3.3%)	311 (4.7%)
Loss OHIP coverage	13 (0.0%)	*3 - 7	*1 - 5	*1 - 5

eTable 3. Reasons for discontinuation and/or censoring among individuals receiving methadone. Treatment discontinuation is operationalized as five outpatient days without dispensation of methadone or previously dispensed take-home doses covering the day.

© 2025 Kleinman RA et al. *JAMA Network Open*.

eFigure 1. Kaplan-Meier curves for time to discontinuation among individuals initiating methadone.

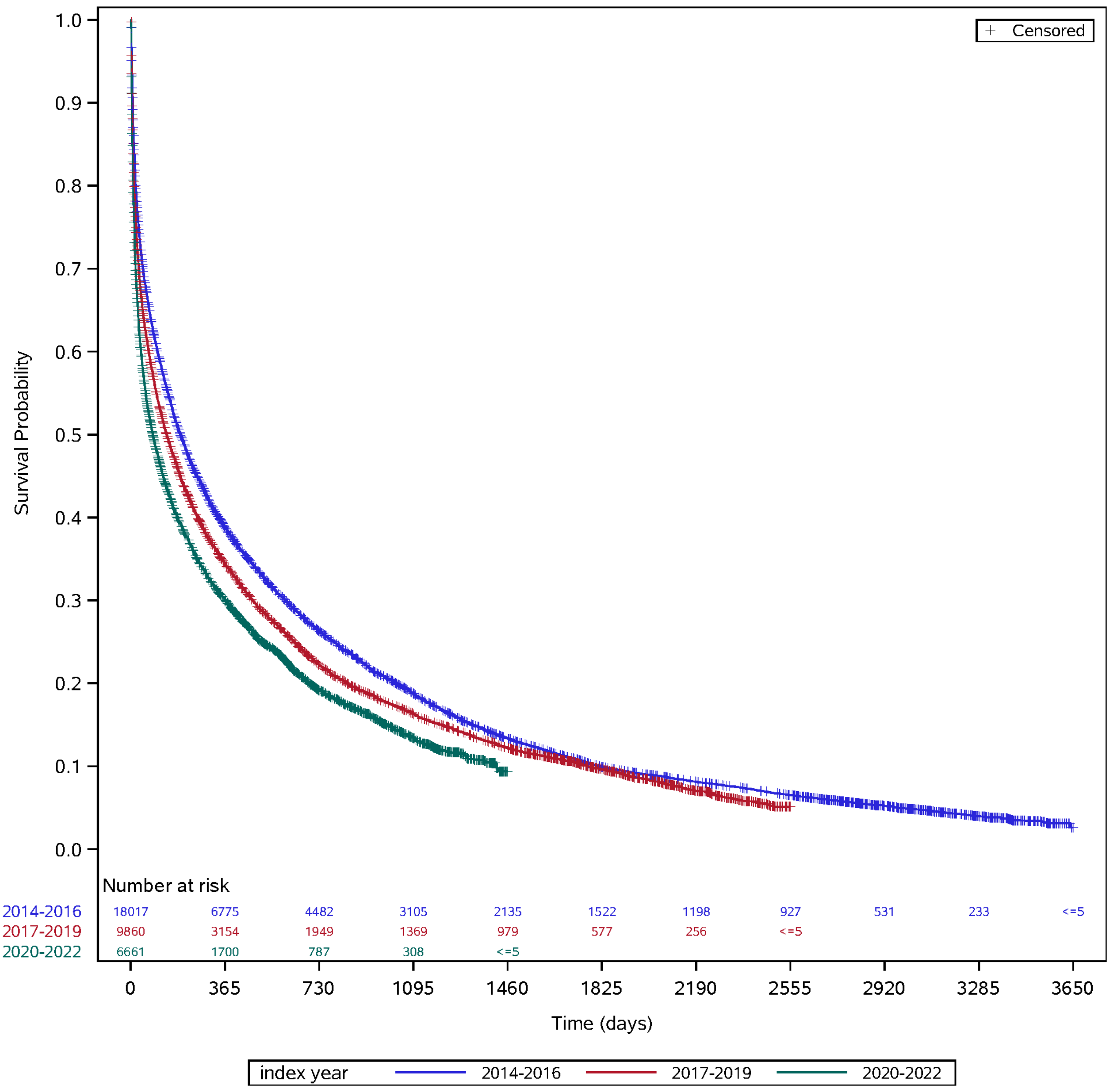

© 2025 Kleinman RA et al. *JAMA Network Open*.

eFigure 2. Log-log plot for adjusted methadone survival model with five-day discontinuation outcome.

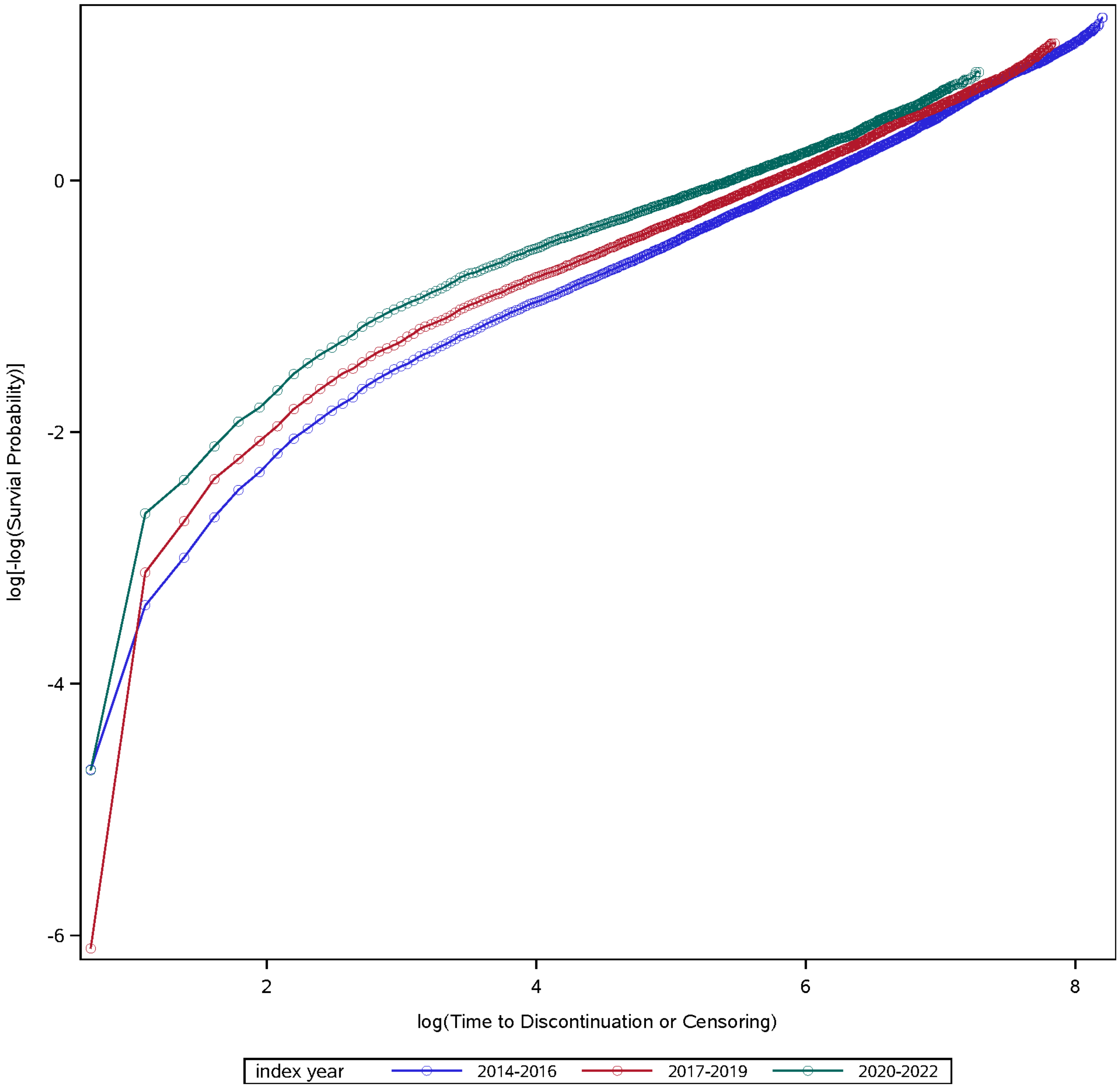

© 2025 Kleinman RA et al. *JAMA Network Open*.

Cox Proportional Hazard Models and Model Diagnostics for primary analysis of buprenorphine-naloxone treatment duration

Covariate	Reference level	Hazard Ratio	P-value
Index period			
2017 – 2019	2014 – 2016	0.98 (0.95-1.00)	0.09
2020 – 2022	2014 – 2016	1.11 (1.08-1.15)	<.001
Time (30 day intervals) by index period interaction			
2017 – 2019		0.99 (0.99-1.00)	<.001
2020 – 2022		0.99 (0.99-0.99)	<.001
Age at index			
15 – 24	35 – 44	1.42 (1.38-1.47)	<.001
25 – 34	35 – 44	1.18 (1.15-1.21)	<.001
45 – 54	35 – 44	0.94 (0.91-0.97)	<.001
55 – 64	35 – 44	0.91 (0.88-0.95)	<.001
65+	35 – 44	1.07 (1.02-1.13)	0.006
Sex			
Male	Female	1.04 (1.02-1.07)	<.001
Rurality			
Rural	Urban	1.11 (1.08-1.14)	<.001
Missing	Urban	1.13 (0.80-1.62)	0.5
Neighbourhood income quintile			
1	5	1.15 (1.12-1.20)	<.001
2	5	1.04 (1.00-1.08)	0.05
3	5	1.04 (1.00-1.09)	0.03
4	5	1.03 (0.99-1.07)	0.1
Missing	5	1.25 (0.89-1.74)	0.2
Johns Hopkins ADGs			
1	0	1.02 (0.97-1.08)	0.4
2	0	1.04 (0.98-1.09)	0.2
3	0	1.05 (1.00-1.11)	0.07
4	0	1.07 (1.02-1.13)	0.009
5	0	1.10 (1.04-1.16)	<.001
6	0	1.09 (1.04-1.15)	0.001
7+	0	1.16 (1.11-1.22)	<.001

eTable 4. Multivariable Cox proportional hazard regression for buprenorphine-naloxone treatment discontinuation. Treatment discontinuation is operationalized as five outpatient days without dispensation of buprenorphine-naloxone or previously dispensed take-home doses covering the day.

© 2025 Kleinman RA et al. *JAMA Network Open*.

Event/censoring	Total	2014-2016	2017-2019	2020-2022
Buprenorphine-naloxone discontinuation	36,518 (95.6%)	11,020 (98.5%)	13,976 (96.7%)	11,522 (91.8%)
Censoring				
End of follow-up (December 31, 2023)	832 (2.2%)	44 (0.4%)	238 (1.6%)	550 (4.4%)
Initiation of buprenorphine-XR	375 (1.0%)	*3 - 7	*47 - 51	321 (2.6%)
Initiation of methadone	268 (0.7%)	88 (0.8%)	100 (0.7%)	80 (0.6%)
Initiation of slow-release oral morphine	*67 - 71	*4 - 8	28 (0.2%)	*31 - 35
Death	112 (0.3%)	20 (0.2%)	56 (0.4%)	36 (0.3%)
Loss OHIP coverage	*3 - 7	0 (0.0%)	*1 - 5	*1 - 5

eTable 5. Reasons for discontinuation and/or censoring among individuals receiving buprenorphine-naloxone. Treatment discontinuation is operationalized as five outpatient days without dispensation of buprenorphine-naloxone or previously dispensed take-home doses covering the day.

© 2025 Kleinman RA et al. *JAMA Network Open*.

eFigure 3. Kaplan-Meier curves for time to discontinuation among individuals initiating buprenorphine-naloxone.

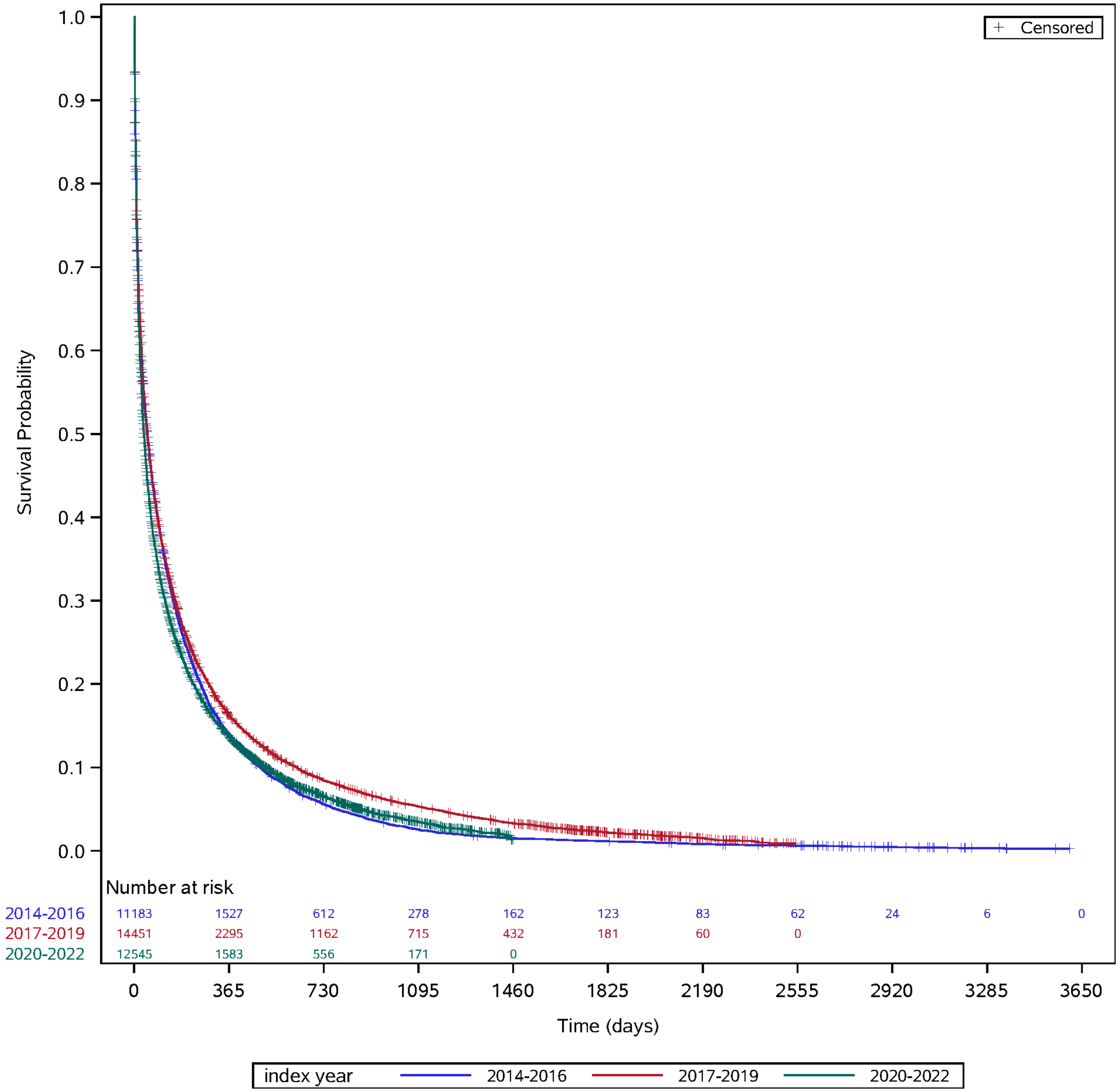

© 2025 Kleinman RA et al. *JAMA Network Open*.

eFigure 4. Log-log plot for adjusted buprenorphine-naloxone survival model with five-day discontinuation outcome.

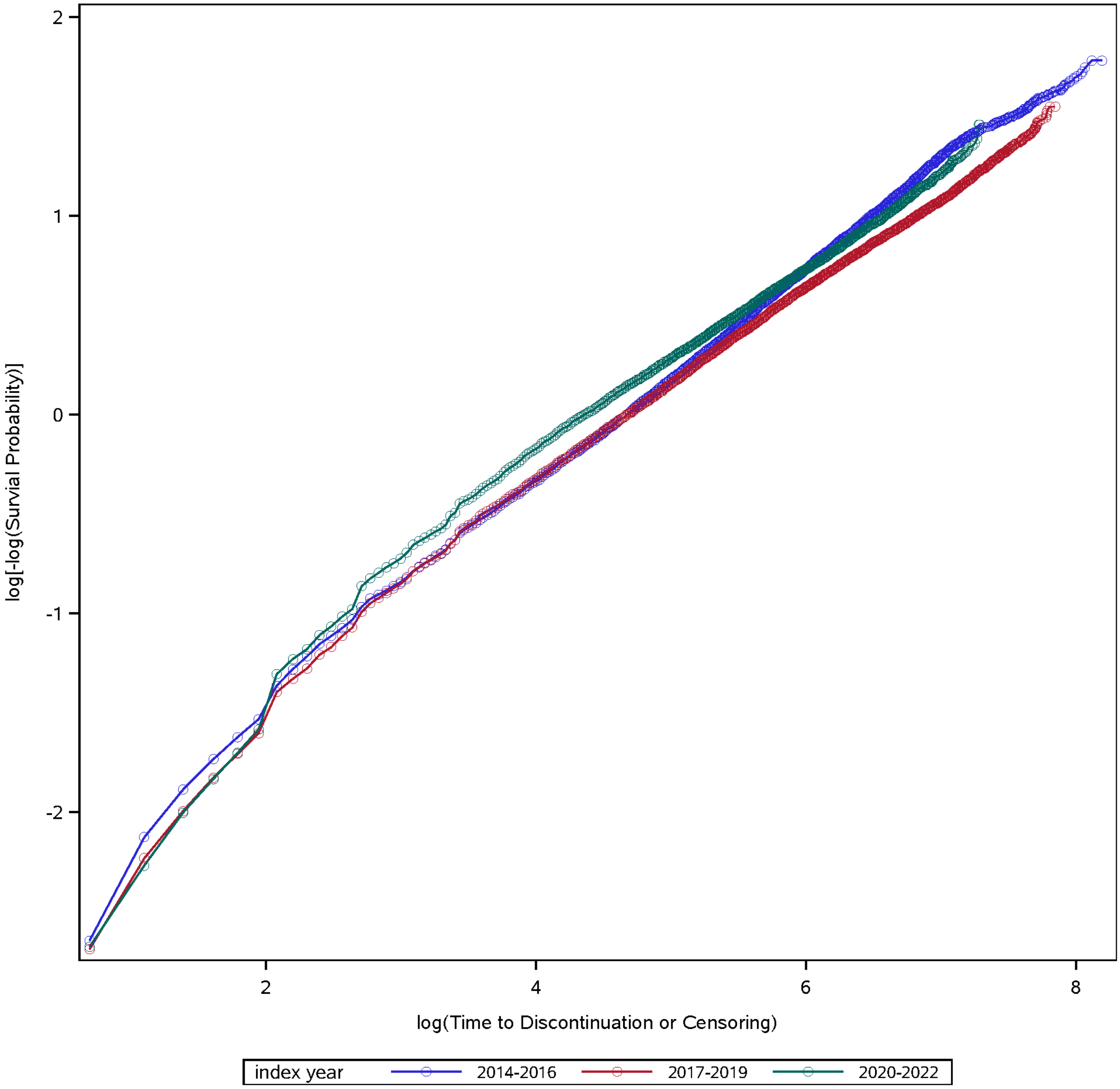

© 2025 Kleinman RA et al. *JAMA Network Open*.

Sensitivity analysis with 14 day period for methadone discontinuation: Multivariable Cox Proportional Hazard Model

Covariate	Reference level	Hazard Ratio	P-value
Index period			
2017 – 2019	2014 – 2016	1.25 (1.20-1.29)	<.001
2020 – 2022	2014 – 2016	1.55 (1.49-1.62)	<.001
Time (30 day intervals) by index period interaction			
2017 – 2019		0.99 (0.99-1.00)	<.001
2020 – 2022		0.99 (0.98-0.99)	<.001
Age at index			
15 – 24	35 – 44	1.35 (1.30-1.40)	<.001
25 – 34	35 – 44	1.20 (1.16-1.24)	<.001
45 – 54	35 – 44	0.88 (0.85-0.92)	<.001
55 – 64	35 – 44	0.79 (0.75-0.83)	<.001
65+	35 – 44	1.43 (1.31-1.56)	<.001
Sex			
Male	Female	1.13 (1.10-1.1.15)	<.001
Rurality			
Rural	Urban	1.02 (0.98-1.05)	0.4
Missing	Urban	2.14 (1.60-1.85)	<.001
Neighbourhood income quintile			
1	5	1.07 (1.03-1.12)	0.001
2	5	1.04 (0.99-1.09)	0.1
3	5	1.00 (0.95-1.05)	0.9
4	5	0.96 (0.91-1.01)	0.1
Missing	5	0.81 (0.62-1.06)	0.1
Johns Hopkins ADGs			
1	0	0.99 (0.94-1.04)	0.6
2	0	1.00 (0.95-1.05)	0.99
3	0	1.04 (0.99-1.09)	0.2
4	0	1.02 (0.97-1.08)	0.4
5	0	1.10 (1.04-1.16)	<.001
6	0	1.12 (1.06-1.19)	<.001
7+	0	1.15 (1.10-1.21)	<.001

eTable 6. Multivariable Cox proportional hazard regression for methadone treatment discontinuation in sensitivity analysis with 14 day period for discontinuation. Treatment discontinuation is operationalized as 14 outpatient days without dispensation of methadone or previously dispensed take-home doses covering the day.

© 2025 Kleinman RA et al. *JAMA Network Open*.

Sensitivity analysis with 14 day period for buprenorphine-naloxone discontinuation: Multivariable Cox Proportional Hazard Model

Covariate	Reference level	Hazard Ratio	P-value
Index period			
2017 – 2019	2014 – 2016	0.95 (0.92-0.98)	<.001
2020 – 2022	2014 – 2016	1.11 (1.07-1.14)	<.001
Time (30 day intervals) by index period interaction			
2017 – 2019		1.00 (1.00-1.00)	<.001
2020 – 2022		1.01 (1.00-1.01)	<.001
Age at index			
15 – 24	35 – 44	1.45 (1.40-1.51)	<.001
25 – 34	35 – 44	1.19 (1.16-1.23)	<.001
45 – 54	35 – 44	0.95 (0.92-0.99)	0.01
55 – 64	35 – 44	0.92 (0.88-0.95)	<.001
65+	35 – 44	1.10 (1.04-1.16)	<.001
Sex			
Male	Female	1.05 (1.02-1.07)	<.001
Rurality			
Rural	Urban	1.07 (1.04-1.10)	<.001
Missing	Urban	1.46 (1.00-2.12)	0.05
Neighbourhood income quintile			
1	5	1.18 (1.14-1.22)	<.001
2	5	1.06 (1.02-1.10)	0.005
3	5	1.03 (0.99-1.08)	0.1
4	5	1.03 (0.98-1.07)	0.2
Missing	5	1.01 (0.71-1.44)	0.9
Johns Hopkins ADGs			
1	0	1.01 (0.96-1.07)	0.6
2	0	1.01 (0.96-1.07)	0.7
3	0	1.03 (0.97-1.08)	0.4
4	0	1.04 (0.98-1.09)	0.2
5	0	1.09 (1.04-1.16)	0.001
6	0	1.09 (1.03-1.15)	0.003
7+	0	1.17 (1.11-1.22)	<.001

eTable 7. Multivariable Cox proportional hazard regression for buprenorphine-naloxone treatment discontinuation in sensitivity analysis with 14 day period for discontinuation. Treatment discontinuation is operationalized as 14 outpatient days without dispensation of buprenorphine-naloxone or previously dispensed take-home doses covering the day.

© 2025 Kleinman RA et al. *JAMA Network Open*.

Sensitivity analysis excluding initiations between March 1, 2019 and February 28, 2021

Time period	Median Treatment Duration, days (95% CI)	
	Methadone	Buprenorphine-naloxone
2014-2016	193 (185 – 202)	51 (49-54)
2017-2019	154 (144-167)	51 (48-53)
2020-2022	81 (71 – 93)	36 (33-38)

eTable 8. Median treatment durations for methadone and buprenorphine in sensitivity analysis excluding initiations between March 1, 2019 and February 28, 2021.

© 2025 Kleinman RA et al. *JAMA Network Open*.

Covariate	Reference level	Hazard Ratio	P-value
Index period			
2017 – 2019	2014 – 2016	1.15 (1.11-1.19)	<.001
2020 – 2022	2014 – 2016	1.52 (1.44-1.60)	<.001
Time (30 day intervals) by index period interaction			
2017 – 2019		1.00 (1.00-1.00)	0.02
2020 – 2022		0.99 (0.98-1.00)	0.001
Age at index			
15 – 24	35 – 44	1.31 (1.26-1.37)	<.001
25 – 34	35 – 44	1.16 (1.12-1.20)	<.001
45 – 54	35 – 44	0.88 (0.84-0.91)	<.001
55 – 64	35 – 44	0.79 (0.74-0.84)	<.001
65+	35 – 44	1.26 (1.14-1.38)	<.001
Sex			
Male	Female	1.09 (1.07-1.12)	<.001
Rurality			
Rural	Urban	1.05 (1.01-1.09)	0.006
Missing	Urban	2.30 (1.70-3.11	<.001
Neighbourhood income quintile			
1	5	1.07 (1.02-1.11)	0.006
2	5	1.04 (0.99-1.09)	0.08
3	5	1.02 (0.97-1.07)	0.5
4	5	0.98 (0.93-1.04)	0.6
Missing	5	0.73 (0.55-0.96)	0.02
Johns Hopkins ADGs			
1	0	1.03 (0.98-1.08)	0.3
2	0	1.01 (0.96-1.06)	0.7
3	0	1.06 (1.01-1.12)	0.03
4	0	1.07 (1.01-1.13)	0.02
5	0	1.09 (1.03-1.15)	0.002
6	0	1.12 (1.05-1.19)	<.001
7+	0	1.18 (1.13-1.23)	<.001

eTable 9. Multivariable Cox proportional hazard regression for methadone treatment discontinuation in sensitivity analysis excluding initiations between March 1, 2019 and February 28, 2021. Treatment discontinuation is operationalized as 5 outpatient days without dispensation of methadone or previously dispensed take-home doses covering the day.

© 2025 Kleinman RA et al. *JAMA Network Open*.

Covariate	Reference level	Hazard Ratio	P-value
Index period			
2017 – 2019	2014 – 2016	0.97 (0.94-1.00)	0.09
2021 – 2022	2014 – 2016	1.18 (1.13-1.22)	<.001
Time (30 day intervals) by index period interaction			
2017 – 2019		0.99 (0.99-1.00)	<.001
2021 – 2022		0.98 (0.98-0.99)	<.001
Age at index			
15 – 24	35 – 44	1.44 (1.38-1.50)	<.001
25 – 34	35 – 44	1.18 (1.14-1.22)	<.001
45 – 54	35 – 44	0.95 (0.92-0.99)	0.02
55 – 64	35 – 44	0.90 (0.86-0.95)	<.001
65+	35 – 44	1.08 (1.02-1.15)	0.01
Sex			
Male	Female	1.04 (1.01-1.06)	0.006
Rurality			
Rural	Urban	1.14 (1.10-1.17)	<.001
Missing	Urban	1.15 (0.75-1.78)	0.5
Neighbourhood income quintile			
1	5	1.16 (1.11-1.20)	<.001
2	5	1.05 (1.00-1.09)	0.04
3	5	1.05 (1.01-1.10)	0.02
4	5	1.02 (0.97-1.07)	0.4
Missing	5	1.26 (0.83-1.90)	0.3
Johns Hopkins ADGs			
1	0	1.06 (0.99-1.12)	0.08
2	0	1.07 (1.01-1.14)	0.02
3	0	1.08 (1.02-1.15)	0.01
4	0	1.09 (1.03-1.16)	0.003
5	0	1.10 (1.03-1.17)	0.002
6	0	1.13 (1.07-1.21)	<.001
7+	0	1.18 (1.12-1.24)	<.001

eTable 10. Multivariable Cox proportional hazard regression for buprenorphine-naloxone treatment discontinuation in sensitivity analysis excluding initiations between March 1, 2019 and February 28, 2021. Treatment discontinuation is operationalized as 5 outpatient days without dispensation of buprenorphine-naloxone or previously dispensed take-home doses covering the day.

© 2025 Kleinman RA et al. *JAMA Network Open*.
