# Supplementary material for: Duration of Methadone and Buprenorphine-Naloxone Treatment
Source: JAMA Netw Open. 2025 Jul 1;8(7):e2518389. doi: 10.1001/jamanetworkopen.2025.18389 (PMC12215571; doi:10.1001/jamanetworkopen.2025.18389)
Supplement: Supplement 2. — Data Sharing Statement [file jamanetwopen-e2518389-s002.pdf]

# Data Sharing Statement

Kleinman. Duration of Methadone and Buprenorphine-Naloxone Treatment. *JAMA Netw Open*. Published July 01, 2025. doi:10.1001/jamanetworkopen.2025.18389

**Data**
**Data available:** No

**Additional Information**
**Explanation for why data not available:** The dataset from this study is held securely in coded form at ICES. While legal data sharing agreements between ICES and data providers (e.g., healthcare organizations and government) prohibit ICES from making the dataset publicly available, access may be granted to those who meet pre-specified criteria for confidential access, available at www.ices.on.ca/DAS (email: das@ices.on.ca). The dataset creation plan and underlying analytic code are available from the authors upon request, understanding that the computer programs may rely upon coding templates or macros that are unique to ICES and are therefore either inaccessible or may require modification.
